# Informality, economic complexity, and internalization of rules

**DOI:** 10.3389/fsoc.2023.1163326

**Published:** 2023-07-14

**Authors:** Marcen Laguna, Iván Hernández, Jesús María Godoy

**Affiliations:** ^1^Department of Economics, Universidad del Rosario, Bogotá, Colombia; ^2^Faculty of Economics and Business, Universidad de Ibagué, Ibagué, Colombia

**Keywords:** informality, self-determination, economic complexity, internalization of institutional rules, labor market

## Abstract

This research aimed to find out the relationship between informality and the internalization of the rules of behavior required for complexity in the economic system, as better knowledge is required for formalization policy to have a greater impact. We use the economic complexity index (ECI) for 2018 at the regional level in Colombia, which combines the country's productive structure with the amount of knowledge and know-how embodied in the goods it produces. The informality measure we use is the individual's affiliation to social security (in particular health insurance), and we use a proxy of civic rule's internalization as an inverse relation with traffic tickets. This research aimed to shed new light on public policy to improve formalization and its economic impact. First, we include a theory that includes both intrinsic and extrinsic motivation types. The self-determination theory or organismic integration theory proposes this theory. Second, we have argued that the motivation to formalize is intrinsic to greater cultural capacity. Individuals gradually internalize rules of behavior that have repercussions on social dynamics. Third, the composition and characteristics of the families in the study sample seem to show that some factors increase the propensity for informality. Our empirical analysis reveals that group of people with a lower educational level are the ones who are more likely to belong to the informal labor market. These results are consistent with the literature. Multivariate Probit regression was used to examine these factors.

## 1. Introduction

The study of informality has gained significant relevance in recent years among different organizations and governments because it is related to economic growth and development (Salazar-Xirinachs and Chacaltana, 2018). Government institutions design different policies to contain and/or understand the role of formality vs. informality (Fernández and Villar, 2017; Boanada-Fuchs and Fuchs, 2018; Salazar-Xirinachs and Chacaltana, 2018). We observe that in the regions where informality is most accentuated, e.g., Latin America, it coincides with regions' facing low levels of economic complexity. Adam et al. (2021) approach this research in the sense of the formal labor market and economic complexity for OECD countries but ignore labor informality. The low levels of economic complexity are an element that explains the disparities in the economic growth of countries and regions (Hausmann et al., 2014; Fritz and Manduca, 2021; Hidalgo, 2021).

Our research is unique in three respects: first, it studies the correlation between informality and economic complexity. Since until now, we have no knowledge of similar investigations. Second, it adds to the research theoretical resources of individual psychology to explain aggregated elements of the economy, and also it theorizes a possible complementarity to the microfoundation on the origin of the preferences of economic agents. For example, this view allows us to understand adaptive learning, just like Uzawa (1968) recursive preferences. In particular, we use the theory of internalization of institutional rules, which to our knowledge has not been considered in economic research. Finally, it contributes to the scarce literature on the study of this triad (i.e., economic complexity, internalization of rules, and informality) at the intra-country level because it provides original results to the literature on labor informality by relating economic complexity with labor informality and internalization of rules of desirable behavior for formalization. The literature gap that we address in this study is the relationship between economic complexity, informality, and the adoption of civic rules as socially desired behavior. In this way, we shed new light on public policies to improve formalization and their economic impact.

A more comprehensive theory is needed for formalization policy to have sustained and lasting effects. Conventional theory has so far focused on extrinsic motivation and neglected intrinsic motivation. A theory that includes both types of motivation is needed (Bénabou and Tirole, 2003). This theory is offered by the self-determination theory in particular the organismic integration theory (Ryan and Deci, 2017). In this sense, a starting point is complex systems. Complex adaptive systems such as society (Holland and Miller, 1991; Hidalgo, 2021) are subject to external and internal interactions, which allow them to change and adapt over time. In the interaction between humans, some elements allow external information to be adopted as one's own (Ryan and Deci, 2017). The study by Welters et al. (2014) coincides with our research in the sense that it addresses a part of the theory of self-determination in the formal labor market once the rules of desirable behavior have been internalized. Consequently, our research is a conceptual extension, since it uses the theory of internalization of rules as a mechanism, and with special attention to labor informality. Therefore, the vision that we defend here is useful because it can serve to generate criteria for policymakers. This research seeks to identify the correlational relationship between labor informality and the internalization of rules of desirable behavior for the greater complexity of the economy and its implications. High economic complexity is desirable because it is associated with higher rates of formal job creation (Lora, 2021).

The structure of the study is as follows. The second section presents a literature review. The third section introduces the theoretical framework. The fourth section shows the empiric strategy, while the last section presents the conclusion.

## 2. Theoretical framework

### 2.1. On subnational complexity and informality

Boanada-Fuchs and Fuchs (2018) synthesize in the taxonomic study of informality an interesting analysis of various investigations associated with understanding informality. It reveals seven dimensions or categories (i.e., economic, legal, technical, organizational, political, social, and cultural) formerly unconnected or unclear, allowing for a transversal understanding of the phenomenon. In the said study, the authors approach formality and informality through a limited choice of publications where the aim is to cover relevant topics.

Similarly, they typify 112 characteristics of informality, which make up a dimension of the previous framework and which are linked to the exposed categories. The following list presents some characteristics that belong to the categories with the greatest and the least participation in the taxonomy they propose, the economic and social. (1) *Economic category*: competitive disadvantage, low wages, rational choice, inefficient, and inequality. (2) *Social category*: self-sufficiency, creativity, family or community-based, short-term strategy, and survival.

In matters of informality, it is also common to distinguish between that which has a labor origin and refers to people from that which has a business origin and involves productive activities. On the one hand, labor informality is associated with low-productivity occupations and labor situations in which workers, voluntarily or involuntarily, are deprived of social rights, social security, and other legal benefits established by the state to improve their working conditions. On the other hand, business informality is related to business registration and operation processes, both from a productive/commercial point of view and from its relationship with the state (Salazar-Xirinachs and Chacaltana, 2018).

Our analysis also considers the four informal sectors from Fernández and Villar's (2017) taxonomy: the informality of subsistence, voluntary, induced, and mixed, as shown in Table 1. Informality is a multidimensional phenomenon in which various economic, structural, institutional, and even political factors intervene, and the processes of globalization, outsourcing, and subcontracting have a powerful influence. Informal, productive entities usually have low organization, with little division between capital and labor. Similarly, informal sector firms have considerably lower productivity than formal sector firms of similar size, and wages and earnings in the informal sector are, on average, lower than in the formal sector (Salazar-Xirinachs and Chacaltana, 2018).

**Table 1:** Taxonomy of informality.

*Subsistence informality* consists of workers who would like to work in the formal sector (or do not have preferences for informality) but find themselves segmented from the labor market because of their low-productivity levels, understood as a combination of education and experience, and place of production. These workers' productivity is well below the level at which a decrease in payroll taxes or adjustments in the legal minimum wage would make any significant difference in their hiring	*Voluntary informality* refers to workers who decide to be informal because they consider that the benefits of informality are greater than those of formal employment. This cost-benefit analysis includes monetary variables, such as income and taxes. However, it also includes other amenities of informality, such as the flexibility of work, the desire not to have a boss, and independence, which are not part of the benefits of formal employment contracts
*Induced informality* comprises workers who are willing to work formally and have the level of productivity needed to be employed widely in this sector but are found relegated to informal jobs because barriers to the formal market can take the form of payroll taxes and other mechanisms of “excessive” protection to the worker. The great limitation at this point is to determine what excessive protection is	*Mixed informality* corresponds to subsistence workers who have very low productivity but express their preference for informality, similarly, in this sense, to voluntary informality

Source: Fernández and Villar (2017).

Research shows that more than half of total employment is informal in most Latin American and Caribbean countries. Although informality provides a life option for a significant percentage of the population, it is also a problem for the region because it mainly affects the vulnerable population. Additionally, it has a fiscal cost because informal workers congest the use of public goods and services without contributing to their financing and because informality can increase corruption (Fernández and Villar, 2017). On a global scale, the International Labor Organization (ILO; Deléchat and Medina, 2021) states that over 60% of the world's adult labor force, or ~2 billion workers, operate in the informal economy.

As mentioned, few studies address the role of economic complexity at the subnational level. An exception is the research by Bandeira Morais et al. (2021), who find that economic complexity is associated with lower income inequality at the subnational level in Brazil. They show that the level of development of the regions affects the effect of economic complexity on income distribution. Similarly, Chávez et al. (2017) found that the ECI is positively associated with a GDP growth rate per capita for Mexico. Additionally, those regions where the population tends to work in more complex activities have, on average, higher levels of GDP per capita.

Hartmann et al. (2017) use multivariate regression analysis to find that economic complexity is a significant and negative predictor of income inequality at the country level and that this relationship is robust to control for aggregate measures of income, institutions, export concentration, and human capital. These findings show that economic complexity captures information about the level of development of an economy that applies to how an economy generates and distributes its income. They also suggest that the productive structure can limit its range of income inequality.

Vu (2020) examines to what extent the combination of products that a country produces (and exports) affects the population's health. The results show that countries with more sophisticated products, on average, enjoy lower mortality rates and higher life expectancies at birth than their counterparts that export less sophisticated products. It argues that the export of a diverse range of complex products is linked to higher income levels, more employment and learning opportunities, better institutions, and equal income distribution within the country. These factors contribute to a better state of national health.

This study aimed to understand the disparities that explain that entrepreneurs are predominantly out of necessity in Colombia, and vice versa, we hypothesize that, in the regions or departments with greater economic complexity, the companies are predominantly by opportunity, between cities and metropolitan areas. Our empirical analysis reveals that departmental contribution to GDP faces disparities of between 16 to 1 between areas such as Bogotá D.C. vs. departments like Caldas and Casanare. Similarly, these disparities are also captured by the sector complexity index (ICS, for its Spanish acronym) provided by DATLAS (https://datlascolombia.bancoldex.com). The ICS is defined as how many productive capacities require for a sector to operate. A sector is complex if it requires a sophisticated level of productive knowledge, such as the financial services and pharmaceutical sectors. Many individuals with different specialized knowledge work in large companies.

It is noteworthy that, in Colombia, the economic structure has become less complex, falling four positions in the ECI ranking in 2019, reaching position 55. In such a way, the growing economic complexity of Colombia has been driven by the lack of diversification of exports, according to the Atlas of Economic Complexity report.^1^ In this sense, the negative relationship between high economic complexity and the lower participation of enterprises by necessity in the productive structure is consistent, given that entrepreneurship out of necessity, driven by motivations and aspirations (Llisterri et al., 2006; Puente et al., 2019), emerges as a solution to unemployment but has no impact on the quality of employment and life.

### 2.2. Relationship between complexity and internalization of rules for formalization

In this research, it is also interesting to understand the role of the diffusion of knowledge (Hidalgo et al., 2018; Coscia et al., 2020), but manifested in aspects such as the internalization of rules, civic values, and desirable behavior for labor formalization, based on the organismic integration theory (OIT) (Welters et al., 2014; Ryan and Deci, 2017). The OIT is the internalization and differentiation of extrinsic motivation and is part of the psychological theories and human behavior that try to explain self-determination. Thus, internalization is how individuals can incorporate or internalize values, beliefs, or behavioral regulations from external sources and transform them into their own (Ryan and Deci, 2017).

The central concepts of the OIT are essentially internalization and integration, which can be understood as a composition of four categories: external regulation, introjected regulation, regulation through identification, and integrated regulation. According to Ryan and Deci (2017), they make up a continuum that exists because those belonging to a social group can adopt behaviors derived from external regulation and whose ultimate result can adopt said regulation as a proper behavior or internalization, generating no internal conflict. Such internalization satisfies their psychological needs for relating to others, competence, and autonomy throughout their lives.

For these reasons, external regulation is necessary but insufficient for cooperation and coordination between individuals. An example of this is the following. A traffic code and sanction mechanisms for offenders are not enough to use a city's vehicular network correctly for private and public users at the local, regional, or national levels. An appropriation or internalization of traffic regulations is required; otherwise, the costs for the use of the road network and the sanctioning mechanism would be prohibitive. That is why there are driving schools and prerequisites, such as obtaining driving licenses, techno-mechanical review, and other requirements related to security measures. These are preventive measures to ensure the proper use of the road network and better coordination and cooperation between users and road authorities.

Therefore, an adequate system of appropriation or internalization leads to a lower level of sanctions, such as, e.g., lower levels of vehicle traffic tickets (ceteris paribus tax pressures). This lower level of sanctions is associated with a greater capacity for coordination and cooperation in mass and flexibility in using the road network at the local, regional, or national level. For these reasons, the variable of traffic tickets is a proxy measure of the greater or lesser capacity for cooperating in a city, region, or country because of the greater or lesser internalization of rules of desirable behavior.

A sustainable transition from the informal to the formal (i.e., sustainable formalization) would be associated with a greater capacity to internalize formal rules. This means a greater ability to internalize rules for desirable behavior in formal companies than informal ones. A correlation between a greater number of traffic tickets and less formal employment is understood partially by very high rates of informal employment in the labor sector (Lora, 2021), therefore, by the non-internalization of rules.

To capture the role of the internalization of rules in formalization, we want to study the internalization of traffic regulations and the reduction in traffic tickets and traffic infractions that have elapsed in Colombia, supplied by the National Federation of Departments. This variable of traffic ticket level is important because it measures the safety of using vehicular transport. It is not a problem but a way to cooperate and coordinate actions massively and flexibly. The internalization of traffic rules is a condition for cooperation and coordination among many people in developing societies. In this sense, the internalization of traffic regulations and compliance with them is desirable (Baker, 1939; Hummel, 2015).

The lower levels of traffic tickets would be measured in the differentials of internalization norms between regions. The word ‘desirable' is mentioned because this type of regulation allows the territories to scale toward more flexible and more extensive cooperation, as is necessary to make the economy more complex—according to authors such as Hidalgo and Hausmann (2009) and Hausmann et al. (2014). In this way, these norms make up a proxy variable for internalizing rules that are transversal to a region, not only those of formalization. With this inclusion, the formalization is studied vis-à-vis regional characteristics of the internalization of established rules for desirable behavior.

Economic complexity measures the amount of productive knowledge each country possesses (Hidalgo, 2021). Similarly, this knowledge is underlying dynamics that could hardly be achieved without cooperation, coordination, and incentives based on interest (e.g., public interest) since economies and societies are complex adaptive systems (Miller et al., 2009; Hidalgo, 2021). Industrial diversification depends on spillover effects from related industries, nearby regions, and their interactions (Gao et al., 2021). Larger cities have higher formal employment rates because creating formal employment is restricted to available skills in complex sectors—producing more sophisticated goods—a path-dependent process (Lora, 2021).

We should note that, from an evolutionary point of view, internalization supports cooperation and cohesive functioning of groups, improving the adaptive advantage at both the individual and group levels of analysis (Boehm, 2012; Ryan and Hawley, 2016). Understanding how to achieve common goals in a society implies understanding the dynamics in which an economy becomes more complex. In particular, how productive knowledge is related to the capacity for the cooperation and coordination of activities? Hausmann et al. (2014) agree that more complex economies have better institutions, more educated workers, and more competitive environments. Similarly, the ECI indirectly captures information on the quality of governance in the countries. In fact, at the company level, formal companies represent this type of diffusion and adoption of established rules to have greater access to capital and technology that make them more productive than small or family businesses (Kumar and Matsusaka, 2009). In Colombia, it has been found that the regions at the subnational level that are most likely to generate formal employment have the greatest economic complexity (Lora, 2021).

In Colombia, Coscia et al. (2017) address the importance of social interactions more than formal institutions^2^ in economic growth. They find evidence of convergence at the municipal level when they belong to a more intense communication cluster.^3^ In contrast, they do not find evidence of a positive influence by the mere fact of belonging to a richer department. Although institutions can vary more between countries than within them for these authors, the diffusion of productive knowledge through networks of intense social interaction can explain why the convergence between locations can be slow and, therefore, the cooperation. Indeed, given that gaining and transferring productive knowledge is costly, if the regions cannot move to productive activities similar to their capacities, they cannot adequately coordinate the accumulation of several new abilities. Hausmann et al. (2014) and Coscia et al. (2017) do not address the issue of the possible regional differential in the internalization of institutional rules for desirable behavior in terms of labor and business formalization.

Countries' labor markets are conditioned by their ‘product space' and, therefore, by the level of sophistication incorporated to produce exported goods (Adam et al., 2021). Similarly, industries that are technologically related to pre-existing industries in a region have a higher probability of entering that region than industries that are not technologically related to pre-existing industries in that region and are related when they require knowledge or similar inputs (Neffke et al., 2011; Hidalgo et al., 2018) and, in fact, the internalization of rules for formalization. Suppose the social agents that allow economic complexity to exist as an emergency cannot coordinate, cooperate, and internalize desirable rules of behavior. In that case, the objectives of labor formality and economic complexity are then translated into higher high rates of informality, precariousness, and low economic growth, since greater economic complexity is correlated with creating formal jobs, and countries that produce more sophisticated products generally have lower rates of unemployment and higher employment rates (Adam et al., 2021; Hidalgo, 2021).

### 2.3. Relationship between the internalization of rules for formalization and informality

In the previous section, we addressed the relationship between economic complexity and the internalization of rules for formalization by incorporating a proxy variable associated with tickets for traffic infringements. However, this same vision is maintained in this section, except to expose its association with informality. The literature recognizes that informality is an important source of income and jobs, but labor activity is considered a problem because of its low impact on economic development (León and Cancino, 2014; Salazar-Xirinachs and Chacaltana, 2018), in such a way that it is important to seek a transition and sustainable development between informality and formality. This change in work activity will be achieved by internalizing established rules for desirable behavior, such as banking that allows greater access to investment credits and, therefore, greater economic growth (World-Bank, 2018). In this way, a region with a significant informal market is expected to have a low internalization of these rules. However, in the literature, it is not named precisely the internalization of rules but an environment that facilitates the inclusion of procedures to register companies and the rule of law (Llisterri et al., 2006). The literature points out the benefits of these procedures but presupposes that they will automatically be internalized as their own if they exist.

Labor informality is subject to multiple mechanisms (Fernández and Villar, 2017; Boanada-Fuchs and Fuchs, 2018; Puente et al., 2019), whose understanding is essential because it provides tools to design adequate policies focused mainly on developing economies (Corbacho et al., 2013; Salazar-Xirinachs and Chacaltana, 2018). Puente et al. (2019) identify a mechanism that allows for explaining changing informality to formality in the Latin American labor market. At first glance, this transition focuses on a psychological aspect (in the aspirations) of the people involved, which is close to the present investigation. Similarly, the economic environment and the internalization of desirable rules (it can be assumed that this internalization can induce aspirations toward formalization) determine the company's subsequent performance during the three or four initial years from the company's beginning. In this way, the differences in human capital in these activities, formal/informal, and therefore the internalization of desirable rules for formalization, are associated with autonomy, competence, and significant work as mechanisms through which it leads to higher levels of wellbeing (Arias and Pena, 2010; Atalay and Tanova, 2022).

In conclusion, because of the high levels of unemployment and informality in Colombia and the lower capacity for mass and flexible cooperation—internalization of traffic regulations is considered as a proxy—the literature shows that in an environment with the existence of procedures, robust institutions, and adequate economic policies, it favors between the internalization of desirable rules, reflected in fewer traffic tickets, and the reduction of informal undertakings. In particular, in countries where the spirit of informality prevails (typically developing countries), there are lower rates of formal business entry (Amorós et al., 2021).

## 3. Literature review

The literature that analyzes the effect of economic complexity on informality is scarce. We can highlight it as a leading and budding current, and it broadens the relationship between formal work and economic complexity (Hidalgo, 2021).^4^

First, Hart (1970) coined the concept of informality in a study of Kenya. In said study, the informal sector was defined as the proportion of employees excluded from the most productive activities, employed in low-productivity activities, and earning low salaries. Similarly, the Deléchat and Medina (2021) defines informal workers as those wage earners whose employment relationship is not attained through labor legislation, the tax system, the social security system, or other labor benefits (compensation for dismissal, vacations, and among others).

Second, The Atlas of Economic Complexity (Hausmann et al., 2014) attempts to measure each country's productive knowledge. In this research, the authors propose a way to measure the capacities and knowledge of the productive sectors that can account for the enormous differences in income between countries and predict the rate at which the countries will grow. Like traditional approaches to economics, economic complexity focuses on the duality between the factors of production, such as labor, capital, and the products that may result from them. However, the economic complexity methods involve detailed data on thousands of economic activities, e.g., exports, to learn about the abstract factors of production and how they are combined into thousands of outcomes (Hidalgo, 2021).

During the last decade, the study of economic complexity as an application to economic growth, income inequality, and among others, has been transferred to the sub-national level in the case of Spain, China, the USA, Brazil, Russia, Mexico, and others (Neffke et al., 2011; Pérez-Balsalobre et al., 2019; Gao et al., 2021; Hidalgo, 2021). It is found that the economic complexity indicators may not capture the spatial heterogeneity in the regions of a country when these differ from their productive structure (Pérez-Balsalobre et al., 2019). Therefore, some metrics of economic complexity have emerged at the subnational level and the international level (Chávez et al., 2017; Lyubimov et al., 2017).

Similarly, Fritz and Manduca (2021) found that the concept of economic complexity translates well from the national to the regional sphere and can incorporate local and commercial industries. They show that economic complexity is well-suited for analyzing the productive structure of regions in the United States. Moreover, their argument proposes that including local sectors is valid and strengthens the analytical power of the complexity indicators. Instead, Gao et al. (2021) approach industrial diversification as dependent on contagion effects from related industries and nearby regions. They focus on the interaction between these two channels, finding that the interaction term is negative and significant, showing that the two spillover channels behave as surrogates.

For a detailed analysis of the productive structure of the countries at the subnational level, the governments of Colombia and Mexico,^5^ together with The Growth Lab-Harvard, carry out in-depth characterizations of these economies. Their objectives are to advance in understanding development challenges and offer viable solutions to reduce global poverty. They serve as a diagnostic tool so that companies, investors, and government authorities can make decisions that help raise the productivity of their respective environments [DATLAS Colombia (http://datlascolombia.com/)]. Additionally, studies have shown that regional economies tend to diversify into sectors related to those already present in the region (Neffke et al., 2011) and that formal and informal institutions influence regional diversification (Cortinovis et al., 2017).

As shown, the theory of economic complexity was proposed by the seminal articles published by Hidalgo et al. (2007) and Hidalgo and Hausmann (2009) and later synthesized in The Atlas of Economic Complexity (Hausmann et al., 2014). The significant role of such research is knowing if the type of products that a country exports matters for subsequent economic performance, thus resulting in an economic complexity index (ECI). Then, the ECI is a measure of the capacity of an economy inferred from the data that connect locations with the activities present within them (Methods-OEC). Economic complexity has also been found to be a useful indicator in other areas. Ahmed et al. (2022) show that in the long run, ECI not only reduces ecological damage but also reduces the adverse environmental impacts of economic growth. Ghosh et al. (2023) found that economic complexity has a significant negative bearing on income inequality, indicating that an increase in structural transformation tends to improve income distribution. They also argue that economies with lower levels of education and less openness to trade fail to reduce inequality in income distribution. Can et al. (2022) reveal that economic complexity increases energy consumption in developing countries while it decreases energy consumption in developed countries. Can and Can (2022) examines the relationship between knowledge and welfare as societal values in an empirical analysis of Turkey. They conclude that the level of knowledge and skills of a society and its level of human welfare move in the long run, and show that the level of knowledge and skills of the society has a positive and statistically significant impact on human welfare in Turkey.

It is helpful to note that Adam et al. (2021) analyze the relationship between the basket of exported products and the labor market. They argue that higher levels of economic sophistication in exported goods lead to lower unemployment and higher employment on the bottom line. It means that moving toward a more complex economy through the development and production of new sophisticated products is a process of creative destruction that directly affects the labor market by creating more jobs than destroying them (Adam et al., 2021).

Similarly, informality has generated significant interest in the labor market, partly because it can absorb part of the destruction of formal jobs after a negative shock (Leyva and Urrutia, 2023). Similarly, in Colombia, the largest cities have higher formal employment rates because creating formal employment is restricted to available skills in complex sectors, a path-dependent process (Lora, 2021).

## 4. Methods and data

**Figure 1:**
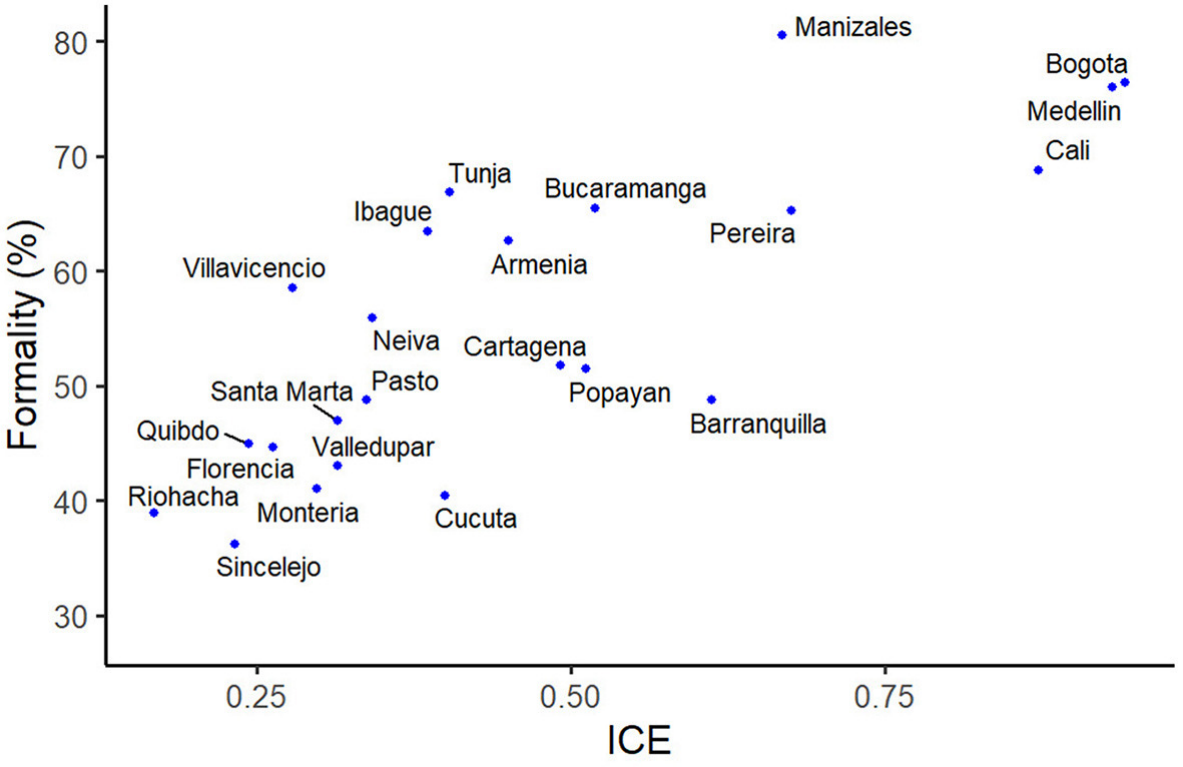
Economic complexity compared to the concentration of formal employment. The ECI is given by DATLAS.

The intuition of our research points to the existence of a mechanism that explains the relationship between productive knowledge, knowledge, and the adoption of prosocial rules on informality. Figure 1 shows that the *a priori* transition goes from knowledge increase to a significant impact on formalization policies.

### 4.1. Strategy econometrics

This study shows the effect of economic complexity and the internalization of desirable behavior rules in the Colombian labor market using the variables described in the previous section through a Probit model. The model was built around a latent regression, as suggested by Hsiao (2014). Specifically, the model used to study the informal labor market is:


(1) $$\begin{array}{l} y_{i}=\alpha_{0}+\beta_{1}ECI_{i}+\beta_{2}Tickets_{i}+\beta_{k}Controls_{i}+\;\varepsilon_{i} \end{array}$$

Here, the response variable *y*_*i*_ takes two values, 1 and 0, to represent whether the individual *i* belongs to the informal labor market or not, respectively, and is expressed as a function of the ECI, which captures the sophistication of the economic structures of a department, the number of traffic tickets per 10,000 inhabitants, a set of exogenous control variables, and a stochastic term ε_*i*_. The model is estimated for three specifications separately to check that the results are robust. The set of exogenous control variables at the individual, family, and national levels, such as gender, age, level of schooling, and economic complexity is consistent with previous research (Hidalgo and Hausmann, 2009; Adam et al., 2021; Amorós et al., 2021).

Suppose that the continuous latent random variable, $y_{i}^{*}$ is a linear function of a vector of explanatory variable, *x*_*i*_, where the error term is ε_*i*_ is independent of *x*_*i*_ with mean 0.


$$\begin{array}{l} y_{i}^{*}=\;\beta^{\prime }x_{i}+\varepsilon_{i} \end{array}$$

What is observable is:


$$\begin{array}{l} y_{i}=\{\begin{array}{ccc} 1, & if & y_{i}^{*}>0,\\ 0, & if & y_{i}^{*}\leq 0. \end{array} \end{array}$$

The, the expected value of *y*_*i*_ is then the probability that the event will occur,


$$\begin{array}{l} E\left(y_{i}|x_{i}\right)=1•Pr\left(\varepsilon_{i}>-\beta^{\prime }x_{i}\right)+0•Pr\left(\varepsilon_{i}<-\beta^{\prime }x_{i}\right) \end{array}$$

$$\begin{array}{l} \;=Pr\left(\varepsilon_{i}>-\beta^{\prime }x_{i}\right)\\ \;=Pr\left(y_{i}=1|x_{i}\;\right). \end{array}$$

When the probability law of generating *vi* follows a two-point distribution $\left(1-\beta^{\prime }x_{i}\right)$ and $\left(-\beta^{\prime }x_{i}\right)$, with probabilities $\beta^{\prime }x_{i}$ and $\left(1-\beta^{\prime }x_{i}\right)$, respectively, we have the linear-probability model. When the probability density function of ε_*i*_ is a standard normal density function, we have the Probit model.

The probabilistic estimates presented here focus on three aspects. The first attempts to address individual determinants. In it, characteristics of individuals such as age, gender, and education are associated. Second, we consider determining factors at the household or family level. For family characteristics, we will consider family size (number of members), social stratum, highest education in the home (regardless of the type of member), dependency in the home (total individuals/total employed), the labor force in the household (labor force/total individuals), and unemployment in the household. Third, we analyze the regional aspect. We specify the base model, along with regional determinants. The size of the labor market in relation to the population (labor force/total population), the unemployment rate in the region, the regional complexity index, the number of traffic tickets as a proxy of the intrinsic motivations to comply with regulations, including formalization, and the universe of individuals (population) in the region.

### 4.2. Economic complexity index

Knowledge is one of the key inputs of production. Because knowledge is stored and shared among people, products are considered vehicles for knowledge transfer and integration (Hausmann et al., 2014). The complexity index is an indicator constructed using international trade data from the United Nations Comtrade database to measure the complexity of a country's economic structures based on the method of reflection (Hidalgo and Hausmann, 2009; see e.g., Hausmann and Hidalgo, 2011; Hausmann et al., 2014).

The complexity of regional economic structures derives from the *diversity* inherent in an economy (the number of products it exports with revealed comparative advantage – RCA), and the *ubiquity* of products (the number of regions that exports a given product with RCA) Hidalgo and Hausmann (2009), then Economic complexity measures the amount of productive knowledge each region possesses 288 (Hidalgo, 2021). The underlying logic is that complex economies are characterized by the ability to produce (and export) a diverse set of sophisticated products with low *ubiquity* because only a small number of regions endowed with many hard-to-find productive capabilities can produce sophisticated goods. By contrast, simpler economies possess limited productive capabilities, thus exporting a less diversified set of *ubiquitous* products Vu (2020).

The measures of *diversity* and *ubiquity* are computed as follows:


$$\begin{array}{l} diversity=k_{R,0}=\overset{N_{p}}{\underset{p=1}{\sum }}M_{Rp}\\ ubiquity=k_{p,0}=\overset{N_{R}}{\underset{R=1}{\sum }}M_{Rp} \end{array}$$

where *R* and *p* stand for region *R* and product *p*. *M*_*Rp*_ equals one if region *R* exports product *p* with RCA, and zero otherwise.

Hidalgo and Hausmann (2009) propose to iterate these equations -reflections method-, then this iterative process starts with the measure of *diversity* *k*_*R*,0_ that captures the number of products a region exports in a given year. Next, *ubiquity* *k*_*p*,0_ is utilized to incorporate information on the ubiquity level of these products. This provides information on the number of products a region exports weighted by the ubiquity of these products. Therefore, in the nth iteration, we get.


$$\begin{array}{l} k_{R,n}=\frac{1}{k_{R,0}}\overset{N_{p}}{\underset{p=1}{\sum }}\;M_{Rp}\;k_{p,n-1} \end{array}$$

where


$$\begin{array}{l} k_{p,n-1}=\frac{1}{k_{p,0}}\overset{N_{R}}{\underset{R=1}{\sum }}\;M_{Rp}\;k_{R,n-2} \end{array}$$

Substituting *k*_*p,n*−1_ 1 into *k*_*R,n*_, we obtain:


$$\begin{array}{l} k_{R,n}=\frac{1}{k_{R,0}}\sum_{p=1}^{N_{p}}M_{Rp}\frac{1}{k_{p,0}}\sum_{R\prime =1}^{N_{R\prime }}M_{R\prime p}k_{R\prime ,n-2} \end{array}$$

As demonstrated by Hausmann et al. (2014), it can be re-expressed as follows:


$$\begin{array}{l} k_{R,n}=\sum_{R\prime }{\tilde{M}}_{RR\prime }k_{R\prime ,n-2}\;in\;which\;{\tilde{M}}_{RR\prime }=\sum_{R\prime }\frac{M_{Rp}M_{R\prime p}}{k_{R,0}k_{p,0}}. \end{array}$$

Finally, the vector of all ECI values for each country is given by:


$$\begin{array}{l} ECI\text{\_}R=\frac{\vec{k}\;-\;\langle \vec{k}\rangle }{std\left(\vec{k}\right)} \end{array}$$

in which $\vec{k}$ is the eigenvector of ${\tilde{M}}_{RR^{\prime }}$ associated with the second largest eigenvalue, as denoted by Hausmann et al. (2014). In addition, 〈□〉 and std stand for average and standard deviation, respectively.

### 4.3. Data

We took the data on the labor market from the Great Integrated Household Survey (GEIH, for its Spanish acronym) of Colombia, and the data on economic complexity are taken from DATLAS (https://datlascolombia.bancoldex.com). Finally, we use the data from the National Federation of Departments to build the proxy variable for the internalization of desirable rules. In this study, following the Deléchat and Medina (2021) and Fernández and Villar (2017), the affiliation to the social security system of people in the condition of work^6^ is defined as an indicator of formality or not.

This study is based on the definition of informality given by Fernández and Villar (2017). Therefore, the variables of interest are informality and formality in the Colombian labor market. For this, we use the data at the individual level for the year 2018, provided by the GEIH because, besides being validated by state entities, it provides a representative sample of the metropolitan areas of Colombia. The GEIH is a survey through which information is requested on people's employment conditions (if they work, what they work for, how much they earn, if they have social security in health, or if they are looking for a job). In addition to the general characteristics of the population, such as sex, age, marital status, and educational level, they are asked about their sources of income.

We use the microdata provided by the National Administrative Department of Statistics (DANE, for its Spanish acronym) in Colombia. It results from the monthly application of a structured questionnaire to a random sample of dwellings in the territory, both in urban and rural areas. The sampling procedures follow all the standard parameters defined by the ILO and are available on the institution's website. The institution visited 228,408 homes in 2018, obtaining information for 766,776 individuals. Of these, 348,015 were occupied according to the criteria defined by DANE.^7^

At the level of individuals, it is identified that there are 324,106 employed individuals, where 93.13% are employed or linked to the social security system in health (p6090). The survey also allows us to distinguish the type of regime to which each individual is affiliated. For the study of informality, we exclude the employed population linked to special regimes (armed forces, Ecopetrol, public universities, and among others) since formalization is automatic in them, and including them will lead to overestimating the labor formalization rate. It is crucial to consider that this research studies the motivations to create a job in the informal sector, and in these regimes, it is feasible to find labor informality.

To study the effect of ECI on labor market informality, we use the data provided by DATLAS, which contains information by department, metropolitan area, and municipality on productive activity, employment, wages, and exports. There are 19 metropolitan areas in the sample, covering 115 municipalities. We also considered that these data present the metropolitan areas and the municipalities with over 50,000 inhabitants, such that at least 75% of its population corresponds to the municipal seat. Table 2 presents a description of the variables that are considered determinants of the formalization aspirations of entrepreneurs, including the dependent variable, which uses a dummy variable where it takes a value of one if it belongs to the informal sector and zero if it belongs to the informal sector. We also control for the level of human capital using the number of people with secondary, technical, and vocational education. Similarly, the ECI quantifies the diversity and sophistication of the export structure, estimated from data that connects the regions of Colombia with the products they export and is freely available in DATLAS. In addition, we obtain traffic tickets from the National Federation of Departments for the year 2018. Finally, we consider the complexity index of 2017, which is merged with the GIEH of 2018 since DATLAS only updated its data up to that year.

**Table 2:** Variables and definitions.

**Variable**	**Definition**	**Source**
ECI	Sector complexity	DATLAS Colombia
Informality	Dummy (=1 if you have social security, =0 if not), question p6090	GEIH
Traffic tickets	Number of traffic tickets per 10,000 inhabitants	Colombian Federation of departments
Population	Number of inhabitants, in millions	DANE
Gender	Dummy (=1 if female, =0 otherwise), question p6020	GEIH
Age	Years of age, question p6040	GEIH
University	Population with undergraduate studies, question p6220	GEIH
Technical or technological	Population with technical or technological studies, question p6220	GEIH
Postgraduate	Population with postgraduate studies, question p6220	GEIH

Table 3 shows the distribution of traffic tickets for the year 2018 and the economic complexity index of the main municipalities of the country for the year 2017. We observe that the more populated areas do not have a higher number of traffic tickets, and the municipalities of Medellin, Barranquilla, and Cali have the highest number of traffic infringements reported. In the same way, the ECI is distributed in a heterogeneous way through these regions, but the ones with the highest score are the ones with the largest number of inhabitants. The municipalities that report the least economic complexity are Villavicencio, Montería, and Pasto. There is heterogeneity between the municipalities with the highest number of traffic infringements and their complexity index.

**Table 3:** Traffic tickets and ECI.

**Municipalities**	**Traffic tickets 2018**	**ECI 2017**
Barranquilla	195.35048	0.61
Bogotá, D.C.	93.97380	0.94
Bucaramanga	92.97231	0.52
Cali	162.03636	0.87
Cartagena de Indias	50.87637	0.49
Ibagué	50.81990	0.39
Manizales	76.06761	0.67
Medellín	203.90676	0.93
Montería	48.38522	0.30
Pasto	118.07956	0.34
Pereira	97.42782	0.68
San José de Cúcuta	66.96922	0.40
Villavicencio	87.65140	0.28

Similarly, Table 4 reports the statistics of the study variables disaggregated at the level of informality and formality. The total of the final sample corresponds to 334,867 individuals, whose average age is between 38 and 39 years for the formal and informal sectors, respectively. Men also predominate in both groups. Within the characteristics associated with human capital, the informal sector group has the largest number of people (73%) with only basic and secondary education, compared to the 45% in the formal sector. On average, the economic complexity of the formal sector is greater (0.49) than that of the informal sector (0.39).

**Table 4:** Statistics.

**Characteristic**	**Formal, *N* = 170,128**	**Informal, *N* = 164,739**
**p6040**		
Age	30 (28, 50)	39 (28, 52)
**p6026**		
Male	89,404 (53%)	95,580 (58%)
Female	80,724 (47%)	69,159 (42%)
**p6220**		
None	1,856 (1.3%)	4,037 (5.5%)
High school	62,453 (45%)	53,276 (73%)
Technical or technological	31,810 (23%)	10,427 (14%)
University	29,643 (22%)	4,687 (6.4%)
Postgraduate	11,860 (8.6%)	341 (0.5%)
Don't know/no response	1 (<0.1%)	2 (<0.1%)
famSiz	4 (3, 5)	4 (3, 5)
famDep	1.67 (1.33, 2.00)	2.00 (1.33, 2.50)
compSec	0.49 (0.34, 0.68)	0.39 (0.30, 0.52)
Tickets10000pop	9.4 (7.6, 16.2)	9.3 (5.1, 16.2)
populationMil	0.97 (0.49, 2.43)	0.71 (0.49, 1.21)

**Figure 2:**
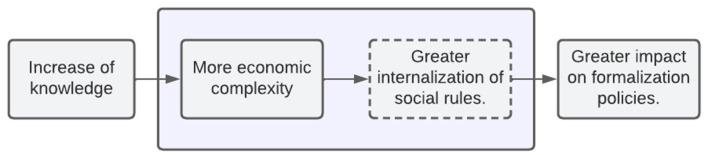
Transmission mechanism.

Figure 2 shows the relationship between the informal sector and the economic complexity of some regions in Colombia. There are more formal enterprises in those places where the economic complexity is greater. Similarly, the economic complexity is very low in areas with a low percentage of formality (Florencia, Sincelejo, Montería, among others). This relationship is consistent with the literature and is not determined by the population size. For example, at the international level, we can see that economic complexity does not correlate with population, showing that it provides a measure of factors driving the geography of activities independent of the population (Hidalgo, 2021).

## 5. Results and discussion

The base model results and its two additional specifications control for individual, family, and regional characteristics. Table 5 reports the results of the Probit estimation. Model 1 reports the probability of belonging to the informal labor market, given individual specifications. It shows that factors such as age or being at a certain level of education have repercussions on the probability of not being an informal worker. Model 2 shows that the size of families, small families, and the smaller number of employed persons within a family increases the propensity to belong to informality.

**Table 5:** Effect of economic complexity and the internalization of desirable behavior rules on labor market informal: Probit regressions.

	**Model 1**	**Model 2**	**Model 3**
(Intercept)	0.86^***^ (0.02)	0.60^***^ (0.02)	1.01^***^ (0.03)
Female (p6020)	0.01 (0.01)	0.01 (0.01)	0.01 (0.01)
Age (p6040)	−0.01^***^ (0.00)	−0.01^***^ (0.00)	−0.01^***^ (0.00)
High school (p6220)	−0.57^***^ (0.02)	−0.56^***^ (0.02)	−0.60^***^ (0.02)
Technical or technological (p6220)	−1.17^***^ (0.02)	−1.15^***^ (0.02)	−1.16^***^ (0.03)
University (p6220)	−1.55^***^ (0.02)	−1.52^***^ (0.02)	−1.51^***^ (0.03)
Postgraduate (p6220)	−2.37^***^ (0.03)	−2.33^***^ (0.03)	−2.25^***^ (0.04)
Don't know/No response	0.05 (0.75)	0.03 (0.75)	3.16 (14.80)
famSiz		0.05^***^ (0.00)	0.04^***^ (0.00)
famDep		0.00 (0.00)	−0.02^***^ (0.00)
compSec			−1.70^***^ (0.03)
Tickets10000pop			0.03^***^ (0.00)
populationMill			0.05^***^ (0.00)
AIC	248,832.43	247,632.69	119,892.04
BIC	248,914.92	247,735.81	120,017.99
Log Likelihood	−124,408.21	−123,806.35	−59,933.02
Deviance	248,816.43	247,612.69	119,866.04
Num. Obs.	222,396	222,396	119,248

*** is significant at the 1% level;

^**^ is significant at the 5% level; ^*^ is significant at the 10% level.

The dependent variable in the three models is labor informality at the individual level explained by three individual variables: sex (p6020: male, female), age in years (p6040), and education (p6040: None, high school, technical or technological, university, postgraduate, does not know/does not report); two family variables: family size (famSiz, number of individuals) and family dependency (famDep = number of people in the household/number of employed persons); and three contextual variables: sector complexity (compSec, sectoral complexity index), number of traffic tickets per 10,000 inhabitants (tickets10000pop), and total inhabitants in the area (populationMill).

This model shows that, as expected, the sign of economic complexity is negative. This coefficient is significant because informal sectors produce low-sophisticated products and, therefore, employ cheap labor with low levels of productive knowledge. The traffic tickets are statistically significant, and their sign is positive, showing that the probability of belonging to the informal sector is associated with low internalization of rules of desirable behavior, therefore, high rates of fines for transit infractions.

When comparing individual characteristics in the three specifications, it is important to point out that educational aspects directly affect the probability of not belonging to the informal labor market. Being in any part of the education cycle, from high school to having a postgraduate degree, increases the probability of belonging to the formality. Similarly, age, whose average is 38 and 39 years 19 for the formal and informal population, respectively, has effects on increasing the probability of being informal. Finally, it is relevant to point out that being a woman implies a moderate disadvantage for informal entrepreneurship in models 1 and 2. However, this effect ceases to be significant in the third specification.

These results fill, in a limited but unique way, the gap in the literature between economic complexity and the labor market, particularly in the understanding of informality. Our results complement the study by Adam et al. (2021), who find a positive relationship between ECI and formal work and a negative relationship between ECI and unemployment. Similarly, this research is related to the self-determination literature. Our results align with Bénabou and Tirole (2003) and Welters et al. (2014), where motivation is essential in coping with the labor market.

Finally, our results do not contradict the literature on economic complexity and desirable behaviors via individual, institutional, or policymakers. As Ghosh et al. (2023), education and social capital are important determinants of good outcomes in reducing inequality. In addition, research associated with economic complexity and its relationship with social welfare (Can and Can, 2022), health and life expectancy (Vu, 2020), even environmental policy management (Ahmed et al., 2022), is also consistent with the study of informality.

### 5.1. Policy implications

Scholars point to the causes and consequences of economic complexity in developed and emerging economies. Research shows that there are heterogeneities between and within countries in productive structures and formal labor rates. Assuming these stylized facts are robust, regional governments should focus on improving productive structures, the quality of education, and creating a collective culture of civic values. As such, these results contribute to the debate on formalization policies in developing economies, e.g., as recommended by the Inter-American Development Bank (Corbacho et al., 2013) and the International Labor Organization (Salazar-Xirinachs and Chacaltana, 2018). These empirical results provide arguments in favor of formalization policy recommendations.

## 6. Conclusion

The following findings suggest that the diffusion of formalization rules, the ability to internalize civic values and productive knowledge (coordination on a larger scale), is subject to difficulties and regional differences of an institutional nature, education, and sex, among others. This research aimed to understand the correlation between informality, economic complexity in Colombia, and the internalization of rules of desirable behavior for labor formalization. This analysis illustrates in first place that regional factors, together with economic complexity, show that informality is less likely in regions with more complex sectors and less export complexity. According to Hidalgo et al. (2007), higher economic complexity is associated with higher productivity. Thus, the underlying mechanism is given by the negative relationship between high economic complexity and informality, as in Lora (2021). Therefore, the results coincide with the evidence of other studies. This result is important and cannot be confused with the stylized fact between the formalization and GDP relationship because economic complexity precisely corrects over- and under-estimation biases that GDP represents, and the loss of direct heterogeneity that this measure presupposes.

Second, we have addressed here that the motivation to formalize is intrinsic to greater cultural capacity. Individuals gradually internalize rules of behavior that have repercussions on social dynamics (i.e., fewer traffic tickets). It is expected that the participants of society adopt progressively institutionally and socially desirable behaviors with the intention that the societies achieve common goals through cooperation. In this way, we establish a variable represented by the variable of traffic infringements, which allowed us to capture the role of the internalization of rules in society considering that the transport system is a common good; this is, therefore, a vision of the social and allows it to be understood as a measure of the common good. Therefore, in no way do they defend any type of stereotypes associated with the culture of poverty and others. This research shows that the probability of the presence of informality is lower when people internalize rules of desirable behavior in the region.

We did not analyze the role of rule internalization in the more complex activities. However, both explanatory variables are included in the informality model since labor informality is not regulated through more autonomous or integrated forms of internalization of rules, especially for the economy's most complex activities (Ryan and Deci, 2017). For this reason, and as shown previously, we found that lower levels of complexity and less internalization of desirable rules of behavior have a positive relationship in explaining the phenomenon of informality. Since societies are complex adaptive dynamic systems, we believe internalization is a feature that can be taken into account by policymakers. This idea supports incentives to create culture, civic values, collective memory, resilience, and promotes the ideal of equity and wellbeing.

Third, the composition and characteristics of the families in the study sample seem to show that some factors increase the propensity for informality. We observe that the group of people with a lower educational level (i.e., secondary school level) are the ones who are more likely to belong to the informal labor market. These results are consistent with the literature. This group of people with basic education is almost twice the size of the group of people with technical and higher education. In addition, the composition of families in the informal sector is those with a very high number of employed persons whose educational level is secondary school. In contrast, in families in the formal sector, the distribution of education levels is very similar between high school and higher education. Therefore, families whose members have basic to advanced education will be less likely to take part in entrepreneurship in the informal market.

### 6.1. Limitations and further research

Our research is subject to limitations. One difficulty that can arise is a bias due to omitted variables. This problem is related to the possibility that relevant factors are excluded from the reference model. A reverse causality could also occur that goes from informality to economic complexity. This reverse causality is ignored by Hartmann et al. (2017) by studying the links between economic complexity, institutions, and income inequality. Similarly, Chu and Hoang (2020) point out the fact that the distribution of a country's income contributes significantly to the accumulation of knowledge. Another challenge is to move from an empirical exercise that is merely exploratory using only cross-sectional data from different subregions of Colombia, to study the temporal dimension of the relationship between economic complexity, internalization of desirable rules, and informality, due to the absence of data over time that allows the analysis of causality. However, since the internalization theory is given, the correlation analysis is sufficient.

There are multiple extensions of this research. For example, in theoretical economics, the application of internalization theory provides insights into the microfoundation of macroeconomic models with heterogeneous agents. Policymakers can also design programs that stimulate collective creativity, and care for public goods and payment of taxes. In behavioral economics, controlled experiments can be designed in which internalization theory can be validated.

## Data availability statement

The raw data supporting the conclusions of this article will be made available by the authors, without undue reservation.

## Author contributions

IH: original draft, methodology, supervising, reviewing the draft, and analysis. JG and ML: methodology, data collection, statistics, software, and reviewing the draft. All authors contributed to the manuscript and approved the submitted version.

## Footnotes

^1^Hausmann et al. (2014).

^2^Formal institutions are understood as municipalities belonging to a particular department. With this, the institutional arrangements are captured.

^3^In particular, Coscia et al. (2017) use mobile phone data to define networks of intense social interaction.

^4^We understand the former from the perspective of macroeconomic and sectoral governance. Consequently, the formal and the informal are perceived as relevant for enumeration, regulation, and taxation. It means that characteristics considered irrelevant from the normative standpoint gain importance and are instead a useful discriminator between the formal and the informal, giving rise to a definition of formal-informal based on the legal (Misra, 2014).

^5^Official government websites, such as Data México from Mexico's Secretary of the Economy (https://datos.gob.mx/complejidad/).

^6^It is noteworthy that in Colombia, decree 806 of 1998 considers an obligation the affiliation and universal coverage of health insurance to the General System of Social Security in Health (SGSSS, for its Spanish acronym) for the entire resident population of the country. Likewise, resolution 3,778 of 2011 emphasizes the uninsured poor population (PPNA, for its Spanish acronym), indicating the mechanisms in which the totality of the affiliation will be carried out.

^7^According to DANE, in Colombia, the population of workers belonging to the informal market corresponds to 48.2% of the workforce for the year 2018.
